# Prognostic Nomogram for Acute Myeloid Leukemia Patients With Biallelic CEBPA Mutations

**DOI:** 10.3389/fonc.2021.628248

**Published:** 2021-08-26

**Authors:** Xiaoyu Xu, Wenzhi Cai, Ping Cai, Ling Zhang, Hong Yao, Tongtong Zhang, Hongjie Shen, Suning Chen

**Affiliations:** ^1^National Clinical Research Center for Hematologic Diseases, Jiangsu Institute of Hematology, The First Affiliated Hospital of Soochow University, Soochow University, Suzhou, China; ^2^Institute of Blood and Marrow Transplantation, Collaborative Innovation Center of Hematology, Soochow University, Suzhou, China; ^3^Department of Hematology, The Affiliated People’s Hospital of Jiangsu University, Zhenjiang, China

**Keywords:** acute myeloid leukemia, biallelic *CEBPA* mutation, prognostic nomogram, *VMP1* expression, CSF3R mutation

## Abstract

Adult acute myeloid leukemia (AML) patients with biallelic mutations of *CEBPA* (bi*CEBPA*) displays a favorable clinical outcome, and is defined as a unique entity in the 2016 World Health Organization classification. However, due to the intrinsic characteristics of the mutation, existence of co-occurring mutations and diversified gene expression signature, the prognosis of these patients needs to be analyzed in a more systematic way. In this study we evaluated the genetic characteristics and clinical outcome in a cohort of 137 bi*CEBPA* AML cases, and proposed a prognostic nomogram to predict the overall survival (OS) of based on the clinical variables selected by multivariate Cox regression model in training cohort, including age, white blood cell count, co-existence of *DNMT3A* and *CSF3R* mutation and whether patients could achieve complete remission after induction therapy. The area under the receiver operating characteristic (ROC) curves for 3 and 5-year OS were 0.833 and 0.863, respectively. RNA sequencing of 4 relapsed patients showed that over-expression of *VMP1* was an indicator of poor prognosis of bi*CEBPA* AML patients. In conclusion, this prognostic nomogram might provide a more accurate prediction of the clinical outcomes of bi*CEBPA* AML patients.

## Introduction

CCAAT/enhancer binding protein α (*CEBPA*) plays a pivotal role as a transcription factor in both self-renewal of hematopoietic stem cells (HSCs) and proliferation and differentiation of myeloid progenitor cells. Major *CEBPA* mutated AML cases carry two mutations, one in the N-terminal of the protein and the other one in the basic leucine zipper (bZIP) domain. N-terminal nonsense and frameshift mutations truncate the *CEBPA* protein and lead to a dominant negative effect, while mutations in the bZIP domain at the C terminus are generally in-frame insertions or deletions which brings disrupted DNA binding and dimerization (1, 2). Biallelic *CEBPA* (bi*CEBPA*) mutations are detected in 2-15% of *de novo* acute myeloid leukemia (AML) patients, and are associated with a favorable clinical outcome compared to wildtype or monoallelic *CEBPA* mutation (3). Due to its biological and clinical significance, AML with bi*CEBPA* mutations has been classified as a distinct entity with an excellent overall prognosis in the World Health Organization (WHO) 2016 edition of classification of tumors of hematopoietic and lymphoid tissues (4–6). However, around 40% patients could relapse after conventional chemotherapy, indicating a blatant heterogeneity within this disease entity (7, 8).

To date, several studies have reported genetic heterogeneity in bi*CEBPA* AML cases, the number of the genes and patients being analyzed was limited. In the present study, we aimed to evaluate the role of concurrent mutations and their prognostic value in bi*CEBPA* AML patients. We will also investigate on underlying reasons of treatment failures in these patients.

## Materials And Methods

### Clinical Patients

From June 2016 to November 2018, a total of 137 *de novo* AML patients who were detected with *CEBPA* mutations and received treatment were enrolled in the study. The diagnosis of these patients fulfilled the criteria of the WHO 2016 edition of myeloid neoplasms and acute leukemia. The study was approved by the Ethics Committee of the First Affiliated Hospital of Soochow University [No. 221 of 2019 LSP (application)] and was conducted following the Declaration of Helsinki. All patients carried biallelic *CEBPA* mutations, involving both the N-terminal TAD1 region and the C-terminal bZIP domain. Major (99/137, 72.3%) patients were treated with standard “3+7” regimen for initial induction therapy (darubicin/idarubicin + cytarabine). In some elderly and severe underlying diseases patients, pre excitation scheme [cytarabine + aclarubicin + granulocyte-colony stimulating factor (G-CSF)] were administered. The first consolidation therapy was generally the same as that used to achieve CR or high/medium-dose cytarabine at 2-3 g/m (2) were administered for consolidation therapy. High-risk patients, or those with a matched sibling, were treated with hematopoietic stem cell transplantation (HSCT).

### DNA Sequencing and Mutation Analysis

Genomic DNA was extracted from bone marrow or peripheral blood samples at the onset of disease diagnosis by using Invitrogen DNA Extraction Kit. The mutational hotspots or whole coding regions of 51 genes (**Supplementary Table 1**) that were recurrently mutated in hematological malignancies were sequenced. The procedures were in accordance with an amplicon-based Next Generation Sequencing (NGS) protocol with Ion Torrent PGM sequencer (Thermo Fisher Scientific, Waltham, MA, USA). An allele frequency threshold of 2% was defined for mutation detection. All *CEBPA* mutations were confirmed by Sanger sequencing. Bone marrow or peripheral blood samples in complete remission (CR) or fingernail samples were also detected for exclusion of *CEBPA* germline mutations.

### RNA Sequencing

RNA was extracted from 16 bone marrow samples of bi*CEBPA* AML cases using TRIzol reagent. These libraries were set up through TruSeq Stranded mRNA LT Sample Prep Kit (Illumina, San Diego, CA, USA). Then these libraries were detected on the Illumina sequencing platform (HiSeq TM 2500 or Illumina HiSeq X Ten) and 125bp/150bp paired-end reads were amplificated.

### Statistical Analysis

Overall survival (OS) is defined as the time from diagnosis to death or to the time of the last follow-up. Disease-free survival (DFS) is defined as the time from CR to relapse、death or the time of last follow-up. All alive patients were followed on December 31, 2019.

The SPSS software (version 23.0; SPSS Inc., Chicago, IL) was applied in statistical analysis. The significance between categorical data was calculated by Chi-square test. Kaplan–Meier method was employed for overall survival analysis, and log-rank test was used to compare differential survival rates between groups. A two-sided P < 0.05 was considered as statistical significance.

## Results

### Clinical Characteristics

The clinical characteristics of patients in the study were summarized in **Table 1** (N=137). The median age of bi*CEBPA* mutated patients in the study was 39.5 years old (range, 10-65 years old), including 82 male and 55 female patients. The median WBC count and PLT count was 21.07×10^9^/L (range, 0.16-384.64×10^9^/L) and 24×10^9^/L (range, 3-431×10^9^/L), while the median bone marrow blast cell percentage was 62% (range, 20.5-90%). Only 32 (23.3%) patients demonstrated abnormal karyotype, among which +21 and del(9q) were the most commonly seen aberrations. In those patients, *GATA2* mutations were the most frequently observed additional mutation, occurring in 21.9% (30/137) patients. Other commonly mutated genes were *WT1* (25/137), *FLT3*-ITD (23/137), *NRAS* (19/137), *CSF3R* (16/137), *C-kit* (14/137) and *DNMT3A* (10/137).

**Table 1:** Clinical characteristics of patients with AML.

Variables	Overall	
	Median	range
Total	137	–
sex		
Male	82	–
Female	55	–
Age (years)	39.5	10-65
WBC (*10 ^9^/L)	21.07	0.16-384.64
HB (g/L)	97	44-163
PLT (*10 ^9^/L)	24	3-431
Blast (%)	62	20.5-90
Karyotype		
normal	105	
abnormal	32	
Induction chemotherapy		
Standard scheme (3 + 7)	99	–
the priming regimen*	38	
One remission failure	13	
Relapse patients	21	–
Transplantation	52	
Death patients	20	–

*low-dose cytarabine and aclarubicin or homo harringtonine in combination with granulocyte colony-stimulating factor.

### Cox Regression Analysis of Training Cohort

**Figure 1:**
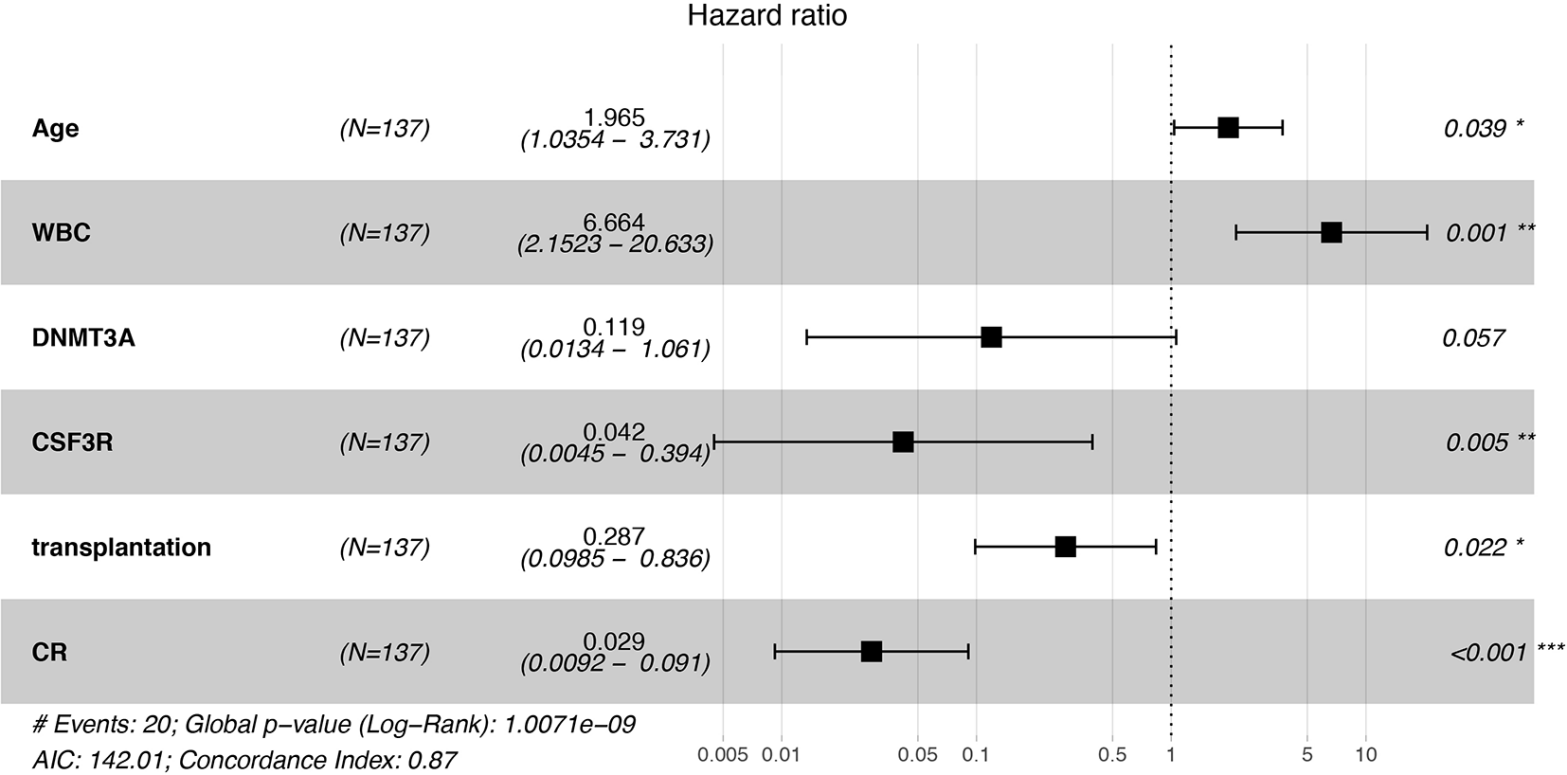
Forest figure for AML patients with bi*CEBPA* mutations. Old age and high WBC count predicted lower overall survival, while CSF3R mutation, transplantation and the achievement of complete remission (CR) was associated with higher overall survival. *p < 0.05, **p < 0.01, ***p < 0.001.

Univariate Cox proportional hazard regression analysis for OS showed that there were significant difference in OS of age, WBC, bone marrow blast cell percentage, one course complete remission and transplantation, which were further included in multivariate Cox regression analysis (**Supplementary Table 2**). *GATA2* mutation, although seen in over 20% of bi*CEBPA* AML cases, did not impact the clinical outcome of these patients (*p* = 0.914, **Supplementary Figure 1**) Multivariate Cox proportional hazard regression models demonstrated that age, whether CR is achieved, transplantation, *CSF3R* mutation were independent prognostic factors for bi*CEBPA* AML (**Figure 1**). We found that *CSF3R* mutation improved the survival of bi*CEBPA* AML patients (*p* = 0.005). In addition, bi*CEBPA* AML patients harboring *DNMT3A* mutation also showed better outcome, although failing to reach statistical significance (*p* = 0.057).

### Nomograms of bi*CEBPA* AML Predicting Survival

**Figure 2:**
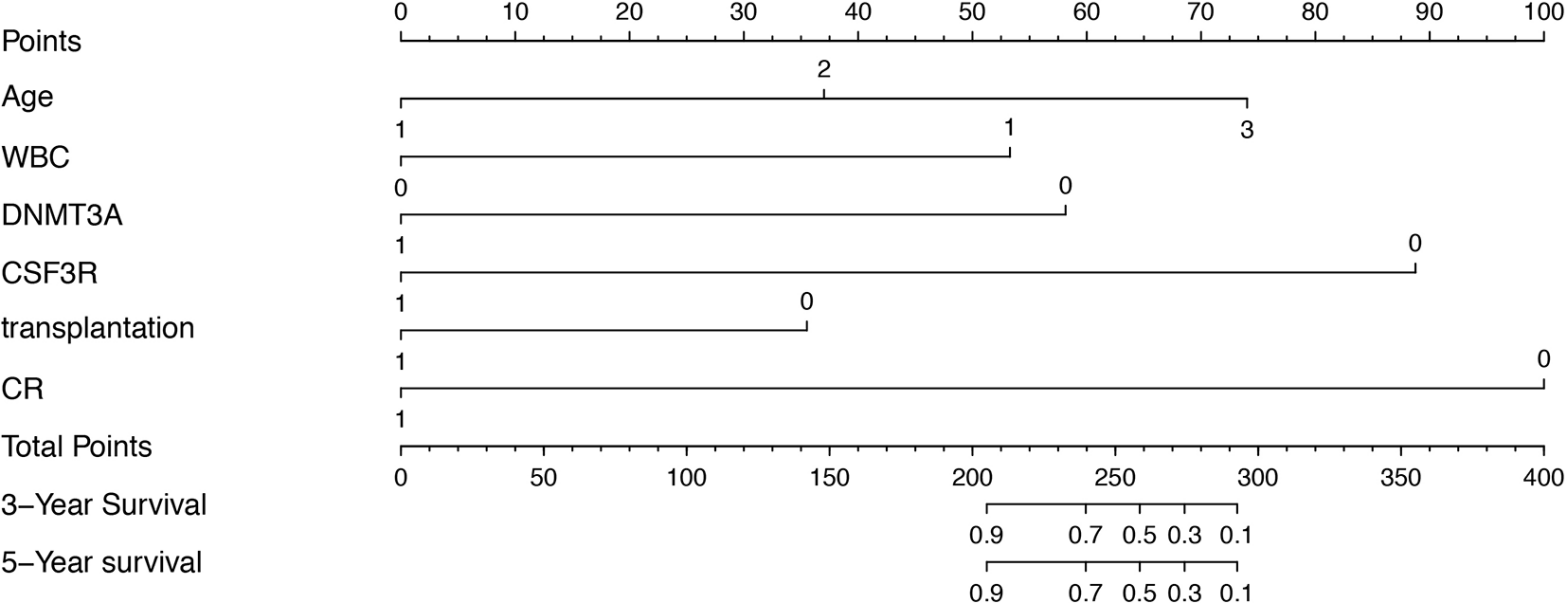
Prognostic nomogram for AML patients with bi*CEBPA* mutations. Age: 1, age < 35 years; 2, age between 35-50 years; 3, age > 51 years. WBC: 1, WBC>25*10^9^/L; 0 WBC<25*10^9^/L. *DNMT3A*: 1, *DNMT3A* mutation positive; 0, *DNMT3A* mutation negative. *CSF3R*: 1, CSF3R mutation positive; 0, *CSF3R* mutation negative. Transplantation: 1, HSCT; 0, non-HSCT. CR: 1, complete remission after one course of induction therapy; 0, failure to achieve complete remission after one course of induction therapy.

Clinical categorical data after multivariate Cox regression were taken into the construction of training cohort nomogram (**Figure 2**). However, due to P < 0.05 of multivariate Cox regression in the OS, WBC and bone marrow blast could not be applied in nomogram.

### Internal Validation

**Figure 3:**
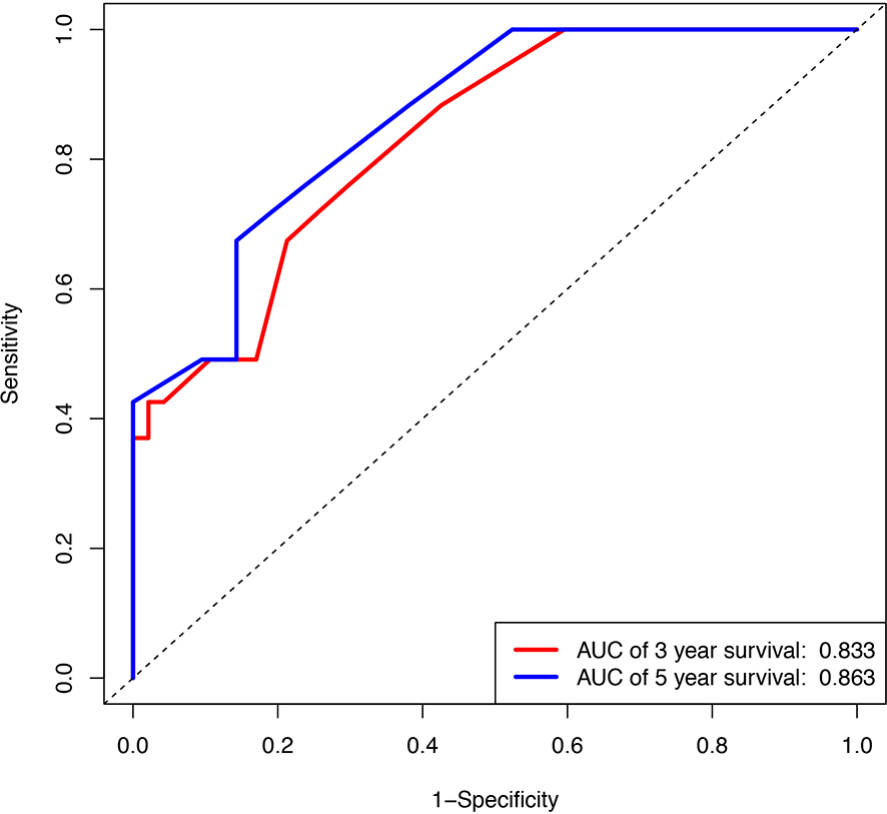
Internal validation of the nomogram to predict OS likelihoods in patients using ROC curve. Blue line, ROC curve of three years survival; Red line, ROC curve of five years survival.

The calibration plot for the probabilities of 3 and 5-year survival rate displayed a great correlation between the actual observed and prediction outcome by this study nomogram (**Supplementary Figure 2**). The predictive ability for OS in training cohort is using ROC curves. The area under the curve (AUC) of ROC curves for 3 and 5-year survival rates were 0.833 and 0.863, respectively (**Figure 3**).

### VMP1 High Expression Predicts Poorer OS

**Figure 4:**
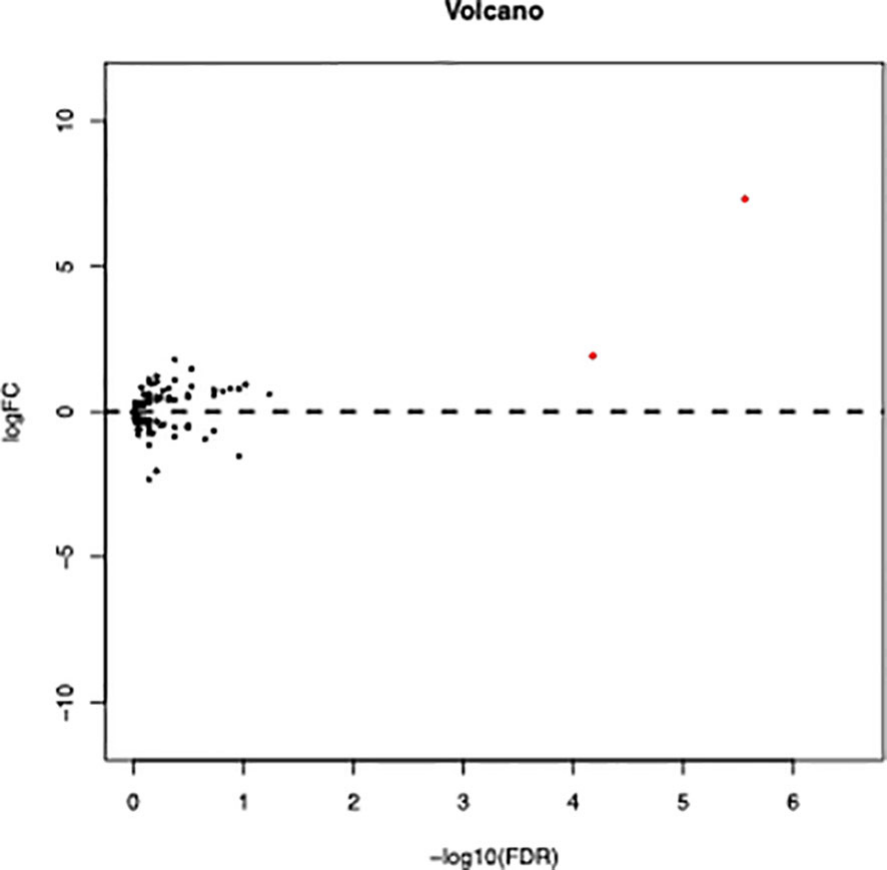
Volcano plot of differentially expressed autophagy related genes between relapse and remission patients with bi*CEBPA* mutation. (FDR < 0.05, *P* < 0.05 and |log2 FC|>1.5).

**Figure 5:**
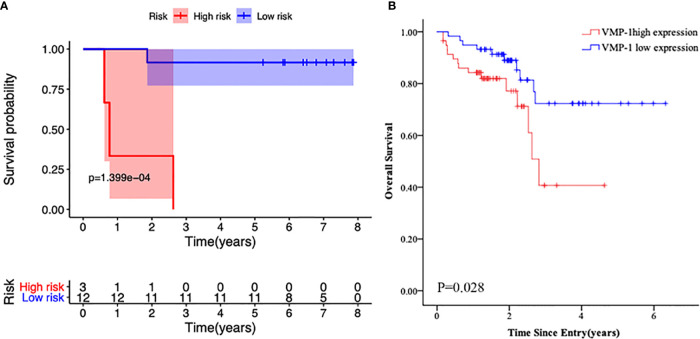
Kaplan-Meier survival curves of the primary cohort according to *VMP1* gene expression. **(A)** Survival probability in bi*CEBPA* AML patients with *VMP1* high vs. low *VMP1* expression. **(B)** Survival probability in normal karyotype AML patients with *VMP1* high *vs.* low *VMP1* expression.

RNA sequencing of samples onset of diagnosis was performed in four patients who finally relapsed as well as 12 patients who survived without detectable genetic aberration. Results showed that autophagy related genes clustered in relapsed samples (**Figure 4**), and the main differences lay in the *NKX2-3* and *VMP1* genes. Furthermore, overexpression of *VMP1* may negatively impact the survival of bi*CEBPA* AML patients (**Figure 5A**, *p* = 0.00014).

To further explore the role of *VMP1* expression in AML patients, we evaluated its expression in another 116 normal karyotype AML patients by real time PCR. We found that high *VMP1* gene expression was an apparent correlation with poor overall survival rate (**Figure 5B**, *p* = 0.028).

## Discussion

Although AML patients with bi*CEBPA* mutation were associated with longer survival, the heterogeneity of these patients was reported in recent years (9). In the present study, we aimed to estimate the probability of 5-year OS based on a multivariate Cox proportional hazards model that included five clinical variables at the onset of diagnosis, including age, white blood cell count, co-existence of *DNMT3A* mutation and/or *CSF3R* mutation and complete remission is achieved after induction therapy.

The best characterized concurrent mutation was *GATA2* mutation. In line with previous studies, we found that *GATA2* mutation, although comprising the highest additional lesion, did not significantly influence the outcome of bi*CEBPA* AML patients showed poor relativity (10–12). Several other mutations such as *FLT3*-ITD (13), *WT1* (14), *CSF3R* mutation (15) are indicators of unfavorable clinical outcome in AML patients with bi*CEBPA* mutations. However, Julia E reported that pediatric AML patients with *CSF3R* mutation had a trend towards low risk disease (p = 0.055) (16, 17). Furthermore, Shigeo Masuda also reported that in bi*CEBPA* AML patients, *DNMT3A* mutations did not impact neither OS nor DFS (18). In our study, *CSF3R* mutation was significantly correlated a better clinical outcome and *DNMT3A* showed a trend. Further studies with a greater number of patients are warranted to test the results.

Relapse is the major cause of treatment failure and final death. In order to probe into the reason why some patients still relapse after remission, RNA sequencing was performed on samples onset of diagnosis in 4 relapsed, with12 patients who survived without detectable genetic aberration as the control. Results showed that the relapsed patients had high expression of blood-related gold and silver *VMP1* gene.

*VMP1* is a transmembrane protein that is related to endoplasmic reticulum, Golgi and intracellular vesicles (19), and is functionally important for cell adhesion, cellular membrane biology and early autophagosome formation (20). It has been shown to be highly expressed in ovarian tumors and is linked to malignant cell proliferation and metastasis (21). Nevertheless, overexpression of *VMP1* gene may decrease the proliferation, invasion and metastasis of tumor cells in colorectal (22) and hepatocellular cancer (23),. This contradiction may be partially explained by different types of tumor, but more importantly, by the role of *VMP1* in autophagy. Hypoxia inducible factors (HIFs) are activated in regions of rapidly growing tumors that are often poorly oxygenated (24). HIF1α expression increases *VMP1*-induced autophagy that results in less cell death in response to photodynamic therapy (25). To date, the impact of *VMP1* in AML is poorly clarified. In this study, we found that bi*CEBPA* AML patients with high VPM1 gene expression had a poor survival, and this may be partially explained by more autophagosome formation, which provides building blocks for cell replication and survival. Taken together, our findings implicated that high *VMP1* expression may be a predictable marker for prognosis of bi*CEBPA*-mutated AML patients.

In conclusion, we found that although AML patients with bi*CEBPA* mutation were generally correlated with an excellent survival, a significant portion of those patients such as those with *VMP1* mutation are still at high risk for relapse. In future studies we will investigate on how *VMP1* mutation will impact the prognosis of bi*CEBPA* AML patients.

## Data Availability Statement

The data presented in the study are deposited in the GSA repository, accession number HDAC000675, https://ngdc.cncb.ac.cn/gsa-human/browse/HRA001135.

## Ethics Statement

The studies involving human participants were reviewed and approved by the Ethics Committee of The First Affiliated Hospital of Soochow University [No. 221 of 2019 LSP (application)] and was conducted following the Declaration of Helsinki. All study participants or their statutory guardian signed informed consent.

## Author Contributions

XX and WC wrote the manuscript. SC developed the treatment concept. XX and PC edited the manuscript and assisted with methods and figures. LZ, TZ, HY, HS, and SC edited the manuscript. All authors contributed to the article and approved the submitted version.

## Funding

This study was supported by grant from the National Key R&D Program of China (2019YFA0111000), the National Natural Science Foundation of China (82000158), the Natural Science Foundation of the Jiangsu Higher Education Institution of China (18KJA320005), the Natural Science Foundation of Jiangsu Province (BK20190180), China Postdoctoral Science Foundation (2018M632372), the priority academic program development of Jiangsu Higher Education Institution, Translational Research Grant of NCRCH (2020WSB11, 2020WSB13).

## Conflict of Interest

The authors declare that the research was conducted in the absence of any commercial or financial relationships that could be construed as a potential conflict of interest.

## Publisher’s Note

All claims expressed in this article are solely those of the authors and do not necessarily represent those of their affiliated organizations, or those of the publisher, the editors and the reviewers. Any product that may be evaluated in this article, or claim that may be made by its manufacturer, is not guaranteed or endorsed by the publisher.

## References

[1] Frohling S Schlenk RF Stolze I Bihlmayr J Benner A Kreitmeier S. CEBPA Mutations in Younger Adults With Acute Myeloid Leukemia and Normal Cytogenetics: Prognostic Relevance and Analysis of Cooperating Mutations. J Clin Oncol (2004) 22(4):624–33. 10.1200/JCO.2004.06.060 14726504

[2] Asou H Gombart AF Takeuchi S Tanaka H Tanioka M Matsui H. Establishment of the Acute Myeloid Leukemia Cell Line Kasumi-6 From a Patient With a Dominant-Negative Mutation in the DNA-Binding Region of the C/EBPalpha Gene. Genes Chromosomes Cancer (2003) 36(2):167–74. 10.1002/gcc.10161 12508245

[3] Wilhelmson AS Porse BT. CCAAT Enhancer Binding Protein Alpha (CEBPA) Biallelic Acute Myeloid Leukaemia: Cooperating Lesions, Molecular Mechanisms and Clinical Relevance. Br J Haematol (2020) 190(4):495–507. 10.1111/bjh.16534 32086816 PMC7496298

[4] Dufour A Schneider F Metzeler KH Hoster E Schneider S Zellmeier E. Acute Myeloid Leukemia With Biallelic CEBPA Gene Mutations and Normal Karyotype Represents a Distinct Genetic Entity Associated With a Favorable Clinical Outcome. J Clin Oncol (2010) 28(4):570–7. 10.1200/JCO.2008.21.6010 20038735

[5] Arber DA Orazi A Hasserjian R Thiele J Borowitz MJ Le Beau MM. The 2016 Revision to the World Health Organization Classification of Myeloid Neoplasms and Acute Leukemia. Blood (2016) 127(20):2391–405. 10.1182/blood-2016-03-643544 27069254

[6] Dohner H Estey E Grimwade D Amadori S Appelbaum FR Buchner T. Diagnosis and Management of AML in Adults: 2017 ELN Recommendations From an International Expert Panel. Blood (2017) 129(4):424–47. 10.1182/blood-2016-08-733196 PMC5291965 27895058

[7] Tallman MS Wang ES Altman JK Appelbaum FR Bhatt VR Bixby D. Acute Myeloid Leukemia, Version 3.2019, NCCN Clinical Practice Guidelines in Oncology. J Natl Compr Canc Netw (2019) 17(6):721–49. 10.6004/jnccn.2019.0028 31200351

[8] Pastore F Kling D Hoster E Dufour A Konstandin NP Schneider S. Long-Term Follow-Up of Cytogenetically Normal CEBPA-Mutated Aml. J Hematol Oncol (2014) 7:55. 10.1186/s13045-014-0055-7 25214041 PMC4172831

[9] Mannelli F Ponziani V Bencini S Bonetti MI Benelli M Cutini I. CEBPA-Double-Mutated Acute Myeloid Leukemia Displays a Unique Phenotypic Profile: A Reliable Screening Method and Insight Into Biological Features. Haematologica (2017) 102(3):529–40. 10.3324/haematol.2016.151910 PMC5394975 28250006

[10] Theis F Corbacioglu A Gaidzik VI Paschka P Weber D Bullinger L. Clinical Impact of GATA2 Mutations in Acute Myeloid Leukemia Patients Harboring CEBPA Mutations: A Study of the AML Study Group. Leukemia (2016) 30(11):2248–50. 10.1038/leu.2016.185 27375010

[11] Green CL Tawana K Hills RK Bodor C Fitzgibbon J Inglott S. GATA2 Mutations in Sporadic and Familial Acute Myeloid Leukaemia Patients With CEBPA Mutations. Br J Haematol (2013) 161(5):701–5. 10.1111/bjh.12317 23560626

[12] Greif PA Dufour A Konstandin NPK sienzyk B Zellmeier E Tizazu B. GATA2 Zinc Finger 1 Mutations Associated With Biallelic CEBPA Mutations Define a Unique Genetic Entity of Acute Myeloid Leukemia. Blood (2012) 120(2):395–403. 10.1182/blood-2012-01-403220 22649106

[13] Green CL Koo KK Hills RK Burnett AK Linch DC Gale RE. Prognostic Significance of CEBPA Mutations in a Large Cohort of Younger Adult Patients With Acute Myeloid Leukemia: Impact of Double CEBPA Mutations and the Interaction With FLT3 and NPM1 Mutations. J Clin Oncol (2010) 28(16):2739–47. 10.1200/JCO.2009.26.2501 20439648

[14] Tien FM Hou HA Tang JL Kuo YY Chen CY Tsai CH. Concomitant WT1 Mutations Predict Poor Prognosis in Acute Myeloid Leukemia Patients With Double Mutant CEBPA. Haematologica (2018) 103(11):e510–3. 10.3324/haematol.2018.189043 PMC6278974 29773598

[15] Su L Gao S Tan Y Lin H Liu X Liu S. CSF3R Mutations Were Associated With an Unfavorable Prognosis in Patients With Acute Myeloid Leukemia With CEBPA Double Mutations. Ann Hematol (2019) 98(7):1641–6. 10.1007/s00277-019-03699-7 31041512

[16] Tarlock K Alonzo T Wang YC Gerbing RB Ries RE Hylkema T. Prognostic Impact of CSF3R Mutations in Favorable Risk Childhood Acute Myeloid Leukemia. Blood (2020) 135(18):1603–6. 10.1182/blood.2019004179 PMC7193184 32187354

[17] Maxson JE Ries RE Wang YC Gerbing RB Kolb EA Thompson SL. CSF3R Mutations Have a High Degree of Overlap With CEBPA Mutations in Pediatric AML. Blood (2016) 127(24):3094–8. 10.1182/blood-2016-04-709899 PMC4911865 27143256

[18] Masuda S. DNMT3A Mutations in Acute Myeloid Leukemia: Impact on Low-Risk Patients With CEBPA Mutations. J Clin Oncol (2011) 29(34):4592–93; author reply 4593-4594. 10.1200/JCO.2011.38.2127 22042957

[19] Tabara LC Vicente JJ Biazik J Eskelinen EL Vincent O Escalante R. Vacuole Membrane Protein 1 Marks Endoplasmic Reticulum Subdomains Enriched in Phospholipid Synthesizing Enzymes and Is Required for Phosphoinositide Distribution. Traffic (2018) 19(8):624–38. 10.1111/tra.12581 29761602

[20] Tabara LC Escalante R. Vmp1 Establishes Er-Microdomains That Regulate Membrane Contact Sites and Autophagy. PloS One (2016) 11(11):e0166499. 10.1371/journal.pone.0166499 27861594 PMC5115753

[21] Zheng L Chen L Zhang X Zhan J Chen J. TMEM49-Related Apoptosis and Metastasis in Ovarian Cancer and Regulated Cell Death. Mol Cell Biochem (2016) 416(1-2):1–9. 10.1007/s11010-016-2684-3 27023910

[22] Guo XZ Ye XL Xiao WZ Wei XN You QH Che XH. Downregulation of VMP1 Confers Aggressive Properties to Colorectal Cancer. Oncol Rep (2015) 34(5):2557–66. 10.3892/or.2015.4240 26328607

[23] Guo L Yang LY Fan C Chen GD Wu F. Novel Roles of Vmp1: Inhibition Metastasis and Proliferation of Hepatocellular Carcinoma. Cancer Sci (2012) 103(12):2110–9. 10.1111/cas.12025 PMC7659295 22971212

[24] Amelio I Melino G. The p53 Family and the Hypoxia-Inducible Factors (Hifs): Determinants of Cancer Progression. Trends Biochem Sci (2015) 40(8):425–34. 10.1016/j.tibs.2015.04.007 26032560

[25] Rodriguez ME Catrinacio C Ropolo A Rivarola VA Vaccaro MI. A Novel HIF-1alpha/VMP1-Autophagic Pathway Induces Resistance to Photodynamic Therapy in Colon Cancer Cells. Photochem Photobiol Sci (2017) 16(11):1631–42. 10.1039/C7PP00161D 28936522

