# Supplementary material for: Prognostic Nomogram for Acute Myeloid Leukemia Patients With Biallelic CEBPA Mutations
Source: Front Oncol. 2021 Aug 26;11:628248. doi: 10.3389/fonc.2021.628248 (PMC8427751; doi:10.3389/fonc.2021.628248)
Supplement: Supplementary file 1 [file DataSheet_1.docx]

**Supplementary Table 1**

| ASXL1 | CEBPA | FLT3 | MPL | PIGA | STAG2 |
| --- | --- | --- | --- | --- | --- |
| ASXL2 | c-kit | GATA2 | MYD88(L265P) | PTEN | TET2 |
| BCOR | CSF3R | IDH1 | NOTCH1 | PTPN11 | TP53 |
| BCORL1 | CSMD1 | IDH2 | NPM1 | RUNX1 | U2AF1 |
| BIRC3 | DNMT3A | IL7R | NRAS | SETBP1 | WT1 |
| BRAF | ETNK1 | JAK1 | PAX5 | SETD2 | ZRSR2 |
| CALR | ETV6 | JAK2 | PDGFRA | SF3B1 |  |
| CBL | EZH2 | JAK3 | PDGFRB | SH2B3 |  |
| CDKN2A(P16) | FBXW7 | KRAS | PHF6 | SRSF2 |  |

**Supplementary Table 2**

| Character | HR | P value |
| --- | --- | --- |
| Age | 1.62241211626607 | 0.097925253645866 |
| WBC  transplantation | 3.38337082149812  0.376338124999572 | 0.0183741745191687  0.0616113096460561 |
| CSF3R | 0.438867725804094 | 0.423003756484573 |
| DNMT3A | 0.674884845751129 | 0.701608673974332 |
| CR | 0.0648550582198389 | 2.47542456322917e-09 |

WBC, white blood cell; CR, complete remission.

**Supplementary Figure 1**


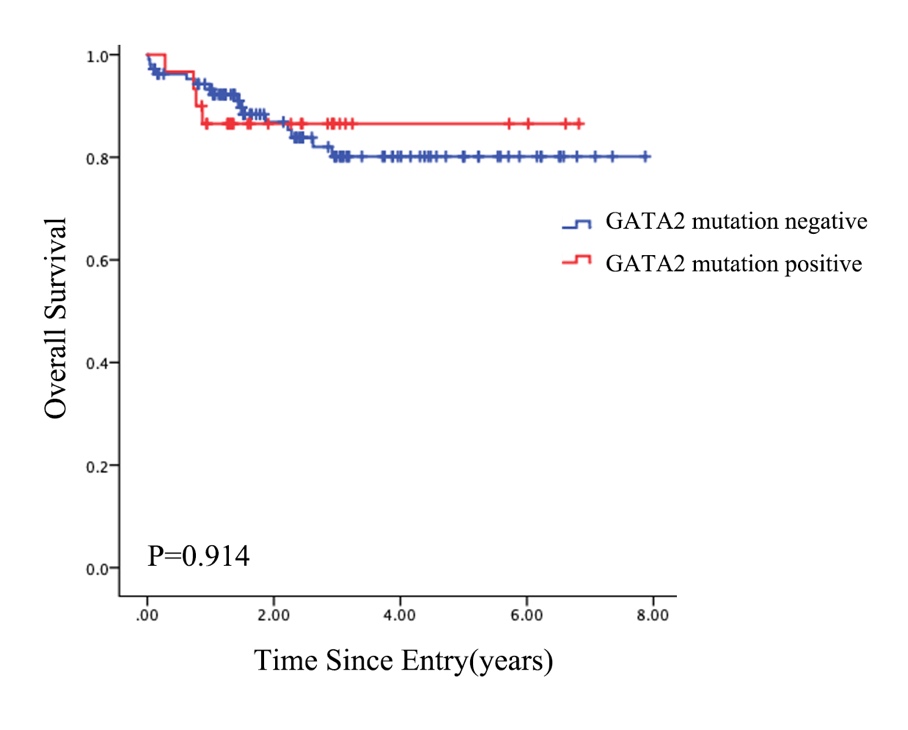


**Figure 1**. Kaplan-Meier survival curves of bi*CEBPA* AML patients according to *GATA2* mutation

*Supplementary Figure 2*

**A B**

**Figure 2**. Internal validation of the nomogram to predict overall survival likelihoods in patients A.3-year survival; B.5-year survival
